# Wnt11 Gene Therapy with Adeno-associated Virus 9 Improves Recovery from Myocardial Infarction by Modulating the Inflammatory Response

**DOI:** 10.1038/srep21705

**Published:** 2016-02-17

**Authors:** Yoshihiro Morishita, Koichi Kobayashi, Ekaterina Klyachko, Kentaro Jujo, Kengo Maeda, Douglas W. Losordo, Toyoaki Murohara

**Affiliations:** 1 Department of Cardiology, Nagoya University Graduate School of Medicine, Nagoya 466-8560, Japan; 2 Feinberg Cardiovascular Research Institute, Northwestern University School of Medicine, Chicago, IL 60611, USA; 3 Tokyo Women’s Medical University, Tokyo 162-0054, Japan; 4 Caladrius Biosciences, Inc. NJ 07920, USA

## Abstract

Acute myocardial infarction induces activation of the acute phase response and infiltration of leukocytes to the infarcted area. Moreover, myocardium that is remote from ischemic area also becomes inflamed. Inflammatory reaction clears dead cells and matrix debris, while prolongation or expansion of the inflammatory response results in dysfunction following myocardial infarction. Wnt glycolipoproteins are best characterized as regulators of embryonic development. Recently several reports suggest that they also contribute to the inflammatory response in adult animals. However, the effects of Wnt proteins on myocardial infarction have not been explored. Here we show that Wnt11 expression leads to significant improvements of survival and cardiac function by suppressing infiltration of multiple kinds of inflammatory cells in infarcted heart. Wnt11 protein suppresses gene expression of inflammatory cytokines through the modulation of NF-κB *in vitro*. These results reveal a novel function of Wnt11 in the regulation of inflammatory response and provide a rationale for the use of Wnt11 to manipulate human diseases that are mediated by inflammation.

Inflammatory response induced by myocardial infarction (MI) is critical for restoring homeostasis in the injured tissue and for activating mechanisms of recovery^1^,^2^. Interleukins are markedly upregulated and, in concert with other cytokines, contribute to the survival and contractility of cardiomyocytes by influencing the recruitment and incorporation of inflammatory cells.^3^ However, sustained inflammation also promotes interstitial fibrosis and collagen deposition in both the area of the infarct and non-infarcted regions of the myocardium^4^,^5^,^6^, which can lead to cardiac rupture or heart failure^5^,^7^. Consequently, the inflammatory response is a key determinant of clinical outcomes for patients recovering from ischemic myocardial injury, and therapeutic approaches that target components of the inflammatory response^8^,^9^ have been investigated as potential and useful treatments for ischemic myocardial injury.

The Wnt glycolipoproteins are best characterized as regulators of cell proliferation, cell polarity, and cell-fate determination during embryonic development; however, components of the Wnt signaling pathway have also been linked to hematological malignancies (e.g., acute and chronic myeloid leukemia, acute lymphoblastic leukemia, multiple myeloma) and to inflammatory diseases such as type 2 diabetes^10^,^11^ in adults. Within the immune system, the canonical Wnt-β-catenin signaling appears to have an important role in the self-renewal of hematopoietic stem cells and progenitor cells, in T- and B-cell development, in peripheral T-cell activation, and in the maturation of dendritic cells^12^,^13^,^14^, whereas non-canonical signaling by Wnt5a may counteract the canonical Wnt signaling to inhibit B- and T-cell development^12^. Wnt5a also regulates the inflammatory response by modulating interleukin production in monocytes and macrophages^15^,^16^. Additionally, Wnt11 which regulates the development of heart and kidney through the non-canonical Wnt signaling suppresses inflammation in intestinal epithelial cells and myocarditis heart^17^,^18^,^19^,^20^. Collectively, these reports suggest that Wnt proteins participate in the inflammatory response and, consequently, that patients recovering from MI might benefit from therapies that alter the Wnt signaling.

Here, we investigated whether the expression of Wnt11, which induces non-canonical Wnt signaling, could alter the inflammatory response and lead to improvements in survival and cardiac function after MI. Genes coding for Wnt11 or LacZ were incorporated into a recombinant adeno-associated virus (rAAV) with single stranded DNA genome and systemically administered to mice 1 week before MI was induced. The rAAV9 vector was chosen because each of the 12 adeno-associated virus (AAV) serotypes (AAV1-AAV12) preferentially tranduces a limited number of tissue types, and the rAAV9 serotype is highly selective for murine cardiac tissue^21^,^22^,^23^. Furthermore, AAVs typically yield long-term gene expression, and more than 40 clinical trials with rAAVs have been approved with no serious adverse events attributable to the vector system^24^.

Compared to the treatment with the rAAV9-LacZ vector, rAAV9-Wnt11 treatment reduced the incorporation of inflammatory cells in infarcted heart and was associated with significantly greater cardiac performance and less fibrosis. Most dramatically, all of the mice treated with rAAV9-Wnt11 survived for the duration of the study, while more than 40% of rAAV9-LacZ–treated mice died during the same period. Moreover, Wnt11 suppressed gene expression of inflammatory cytokines from stimulated macrophage cell line, accompanied by the regulation of NF-κB. Our study revealed that Wnt11 expression significantly improves recovery from ischemic myocardial injury by reducing the inflammatory response.

## Results

### Systemic rAAV9 administration leads to persistent, cardiac-specific gene expression

**Figure 1:**
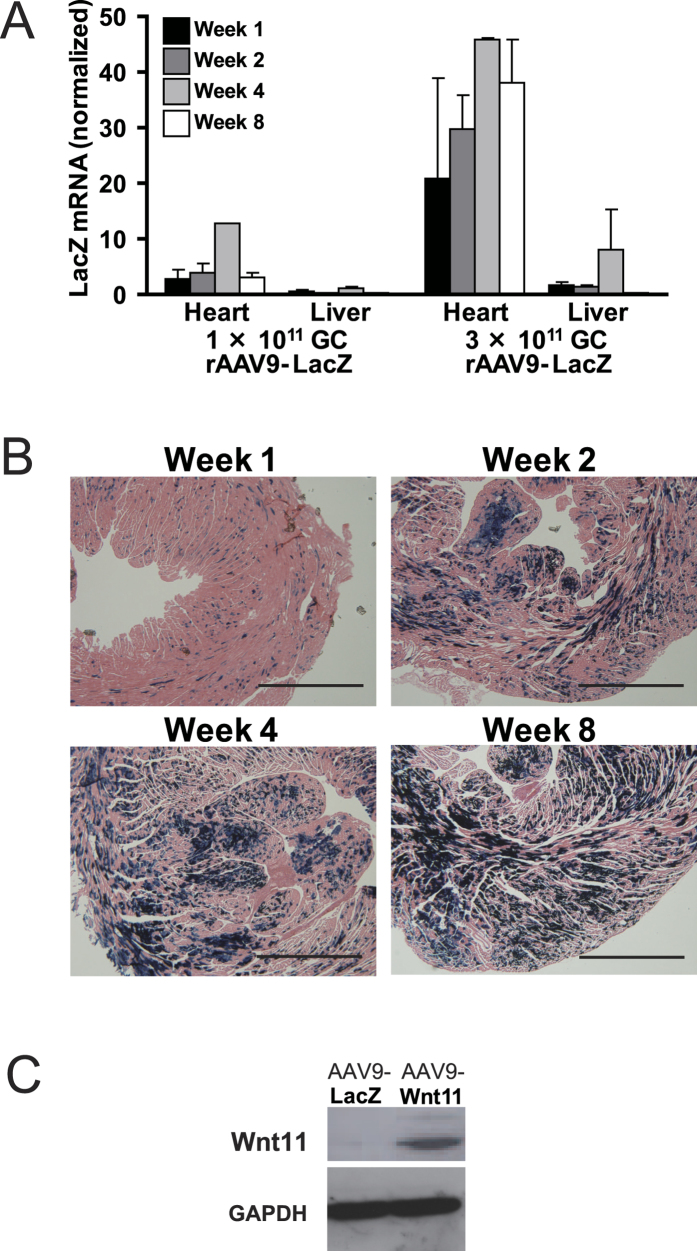
The rAAV9 Vector Induces Robust, Durable, and Cardiac-specific Gene Expression. (**A**) 1.0 × 10^11^ GC or 3.0 × 10^11^ GC of an rAAV9 vector coding for LacZ expression was injected into the tail vein of uninjured mice, and LacZ mRNA expression in the heart and liver was monitored for 8 weeks using quantitative RT-PCR. LacZ expression was normalized to 18S rRNA expression; n = 2 per treatment group. (**B**) LacZ expression was identified in the hearts of mice sacrificed 1–8 weeks after the treatment by 3.0 × 10^11^ GC of an rAAV9 vector coding for LacZ expression via X-gal staining; scale bar = 1 mm. (**C**) 3.0 × 10^11^ GC of an rAAV9 vector coding for Wnt11 expression or LacZ was injected into the tail vein of uninjured mice, and Wnt11 protein expression was evaluated 2 weeks later via Western blot.

The efficiency, duration, specificity, and dose-dependence of rAAV9-mediated transduction were evaluated by injecting rAAV9-LacZ into the tail vein of uninjured mice and monitoring LacZ expression up to the next 8 weeks. LacZ mRNA was abundantly expressed in the heart throughout this period and was markedly higher in mice injected with 3.0 × 10^11^ GC of the virus than in mice administered a 1.0 × 10^11^-GC dose (Fig. 1A). LacZ mRNA expression was also observed in the liver, but at much lower levels, while expression in the kidney, bone marrow, pancreas, lung, brain, intestine, spleen, and testes was nearly undetectable. X-gal-stained sections of cardiac tissue indicated that cardiomyocytes, but no other cell type, expressed LacZ in the heart (Fig. 1B), and that a substantial proportion of cardiomyocytes expressed the transduced gene within two weeks of injection. To confirm that Wnt11 could be transduced with the rAAV9 vector, Wnt11 protein levels were evaluated in cardiac tissues of mice injected with 3.0 × 10^11^ GC of rAAV9-Wnt11; high Wnt11 protein level was observed 2 weeks after injection (Fig. 1C). We also examined endogenous expression of Wnt11 gene after MI induction without rAAV9 injection. Wnt11 expression was not enhanced in infarcted heart (Suppl. Fig. 1).

### Systemic rAAV9-Wnt11 administration improves survival and cardiac performance after MI in mice

**Figure 2:**
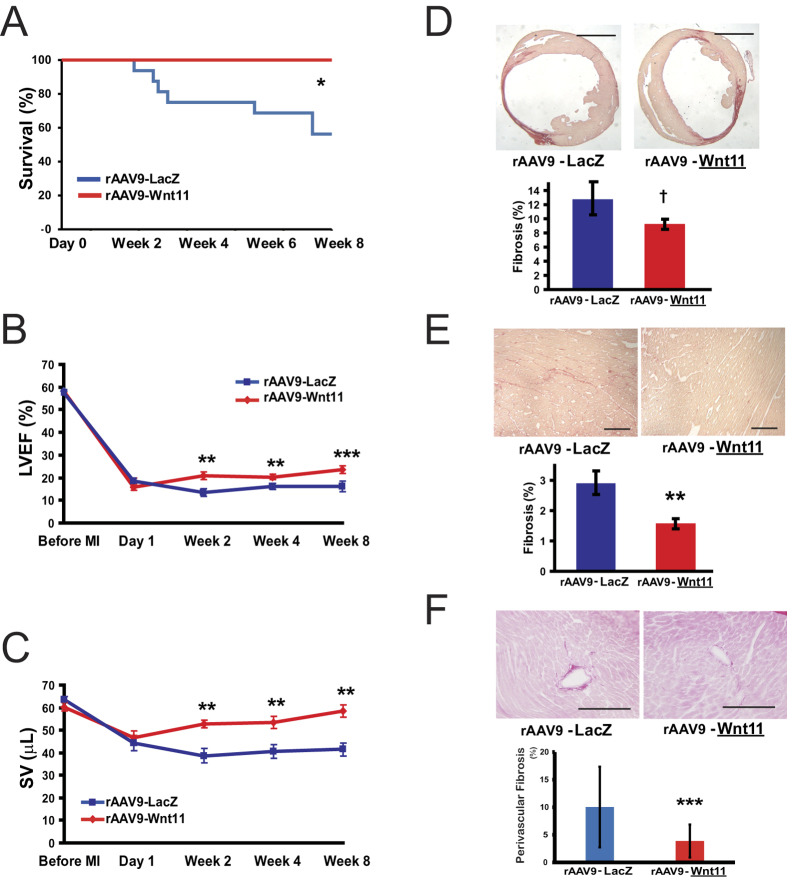
Wnt11 Expression during Recovery from MI Improves Survival and Cardiac Function and Reduces Fibrosis in Non-infarcted Regions of the Heart. One week before surgically induced MI, 3.0 × 10^11^ GC of an rAAV9 vector coding for Wnt11 (rAAV9-Wnt11) or LacZ (rAAV9-LacZ) expression was injected into the tail vein of wild type (WT) mice transplanted with bone marrow from GFP-expressing mice. (**A**) Survival was monitored for 8 weeks after MI; n = 16 per treatment group at day 0. *P < 0.01. Echocardiographic assessments of (**B**) left-ventricular ejection fraction (LVEF) and (**C**) stroke volume (SV) were performed before injury and repeated in surviving mice on day 1 and weeks 2–8 after MI. rAAV-Wnt11: n = 16 at all time points, rAAV9-LacZ: n = 16 Before MI and at Day 1 and Week 2; n = 12 at Week 4; and n = 9 at Week8. **P < 0.005; ***P < 0.0001. Fibrosis was evaluated in Sirius-red-stained sections of tissue harvested from the (**D**) whole heart (scale bar = 2 mm) and from the (**E,F**) non-infarcted region (scale bar = 100 μm) 8 weeks after MI induction. Fibrosis (**D,E**) and perivascular fibrosis (**F**) were quantified as the ratio of the area of fibrosis to the total area and the ratio of the area of fibrosis to the total vessel area, respectively. rAAV9-LacZ: n = 9; rAAV9-Wnt11: n = 16. ^†^*P* = 0.065; **P < 0.005; ***P < 0.05.

Potential benefit of Wnt11 gene expression during recovery from ischemic cardiac injury was evaluated by injecting 3.0 × 10^11^ GC of rAAV9-Wnt11 or rAAV9-LacZ into the tail vein of WT mice transplanted with bone marrow from GFP-expressing mice one week before surgically induced MI. MI was induced by ligating the proximal site of the LAD coronary artery, thereby inducing larger-sized infarcts that typically lead to a relatively high mortality rate. Nevertheless, all mice administered rAAV9-Wnt11 survived until sacrifice at week 8, while more than 40% of mice treated with rAAV9-LacZ died during the same period (p < 0.01) (Fig. 2A). Autopsy examinations found evidence of pleural effusion in the deceased mice, suggesting that the cause of death was heart failure.

To determine whether rAAV9-Wnt11 treatment enhanced cardiac function after ischemic injury, echocardiographic assessments of left-ventricular ejection fraction (LVEF), Left ventricular end diastolic volume (LVEDV) and stroke volume (SV) were performed before injury and repeated in surviving mice on day 1 and weeks 2, 4, and 8 after MI. LVEF (Fig. 2B), SV (Fig. 2C) and cardiac output (Suppl. Fig. 2A) were significantly higher (p < 0.005) in mice administered rAAV9-Wnt11 than in rAAV9-LacZ-treated mice at all weekly time points. These significant differences cannot be attributed to mortality in the rAAV9-LacZ-treatment group, because mean LVEF and SV was higher at weeks 2 and 4 in the surviving rAAV9-LacZ-treated mice than in those that subsequently died. On the other hand, Wnt11 expression does not significantly change left ventricular end-diastolic volume (LVEDV) and left ventricular end-systolic volume (LVESV), which showed a tendency to decrease in rAAV9-Wnt11 treated mice (Suppl. Fig. 2B,C).

### Systemic rAAV9-Wnt11 administration reduces fibrosis in non-infarcted regions of the heart after MI

The potential influence of Wnt11 gene expression on infarct size was evaluated by measuring the extent of fibrosis in Sirius-red-stained sections from the hearts of mice that survived through week 8. Fibrosis was notably less extensive in rAAV9-Wnt11-treated mice than in mice treated with rAAV9-LacZ, but the difference between the groups did not reach statistical significance (p = 0.065) (Fig. 2D). However, in the non-infarcted regions, fibrosis and perivascular fibrosis was significantly lower after treatment with rAAV9-Wnt11 than after rAAV9-LacZ treatment (Fig. 2E,F).

Thus, a considerable proportion of the benefit associated with Wnt11 gene expression during myocardial recovery may be attributable to improvements in non-infarcted regions of the heart.

### Systemic rAAV9-Wnt11 administration exclusively suppresses the incorporation of bone-marrow-derived cells and inflammatory cells after MI

**Figure 3:**
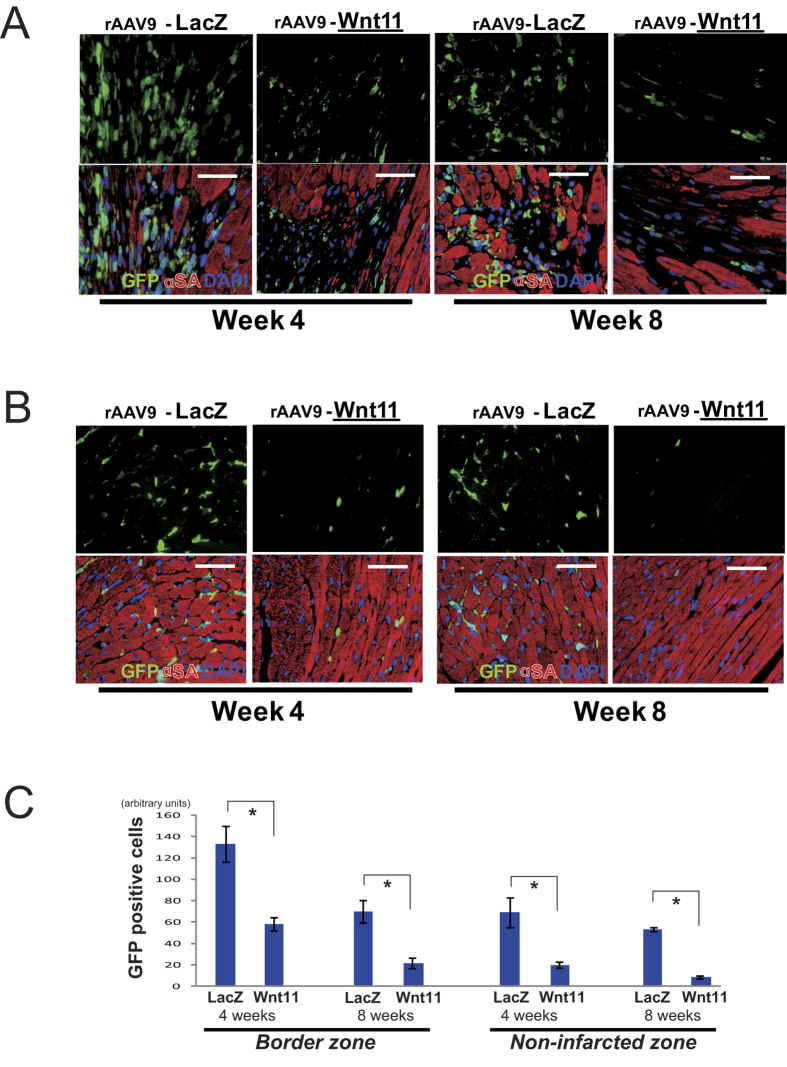
Wnt11 Expression during Recovery from MI Reduces the Incorporation of Bone-marrow–derived Cells. Four and 8 weeks after MI, mice were sacrificed, and bone-marrow derived (i.e., GFP+) cells were identified in the (**A**) border-zone of the infarct and in the (**B**) non-infarcted region by staining sections for GFP (green) and α-sarcomeric actin (αSA, red); nuclei were counter-stained with DAPI (blue); scale bar = 100μm. (**C**) GFP positive cells in the border or non-infarcted region of each group were quantified; n = 4 per each group. *P < 0.05.

**Figure 4:**
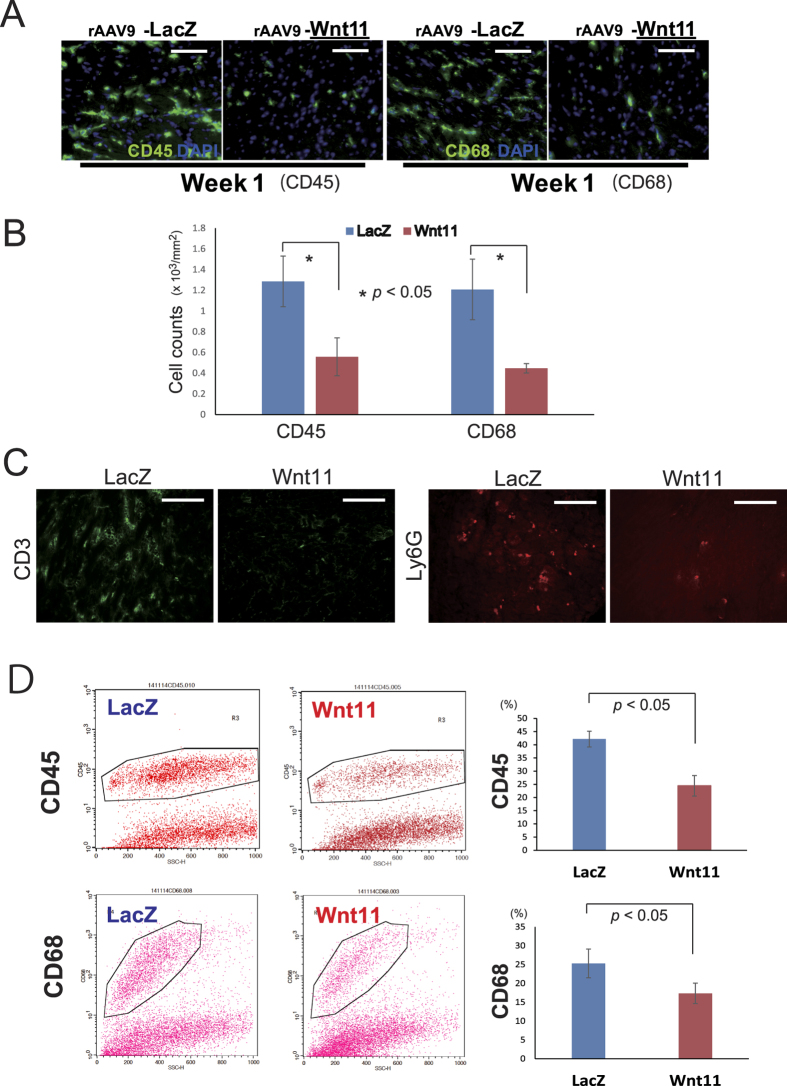
Wnt11 Expression during Recovery from MI Reduces the Incorporation of Inflammatory Cells. (**A**) One week after MI, mice were sacrificed, and inflammatory cells were identified in the non-infarcted region by staining sections for expression of the inflammatory-cell markers CD45 (green, left panels) or CD68 (green, right panels). Nuclei were counter-stained with DAPI; scale bar = 100μm. (**B**) CD45 or CD68 positive cells in the non-infarcted region of each group were quantified; n = 3 per each group. *P < 0.05. (**C**) CD3 (T cell antigen, green) and Ly6G (neutrophil antigen, red) positive cells were evaluated in the heart tissue 1 week after MI. Scale bar = 100 μm. (**D**) CD45 or CD68 positive cells in infarcted heart tissue on day 7 were evaluated by fluorescence activated cell sorting (FACS) after digestion by collagenase and dispase. Ratio of CD45 or CD68 to total cell number was quantified; n = 3 per each group.

Because cells mobilized from the bone marrow participate in the inflammatory response, MI was induced in WT mice transplanted with bone marrow from GFP-expressing mice, and bone-marrow-derived (i.e., GFP-positive) cells were identified in the hearts of mice sacrificed 4 and 8 weeks later. Bone-marrow-derived cells were notably less common in hearts from rAAV9-Wnt11-treated mice than in hearts from mice treated with rAAV9-LacZ, both in the border zone of the infarct (Fig. 3A,C) and in non-infarcted regions (Fig. 3B,C). Furthermore, sections stained for expression of the inflammatory markers CD45 (leukocyte common antigen) and CD68 (macrophage antigen) indicated that 1 week after MI, rAAV9-Wnt11 administration was significantly associated with fewer inflammatory cells in non-infarcted cardiac tissue (Fig. 4A,B). Surprisingly, CD3 (T cell antigen) and Ly6G (neutrophil antigen) positive cells are also decreased by Wnt11 expression in non-infarcted cardiac tissue 1 week after MI (Fig. 4C). Thus, Wnt11 gene expression during recovery from MI appears to reduce the incorporation of bone-marrow-derived cells and multiple subsets of leukocytes.

Moreover, we evaluated infiltration of inflammatory cells in infarcted heart on day 7 by fluorescence activated cell sorting (FACS) analysis. The ratio of CD45 and CD68 positive cells to isolated cells from whole heart was significantly lower in rAAV9-Wnt11-treated mice than in rAAV9-LacZ-treated mice (Fig. 4D).

Wnt11 is reported to regulate cardiac differentiation in embryonic development. Bone marrow derived cells are the most important candidate which may differentiate into cardiomyocytes by Wnt11. Cardiac differentiation of bone marrow derived cells were evaluated by double staining of GFP (bone marrow derived cells) and α-sarcomeric actin (α-SA). Double positive cell of GFP and α-SA was not found in all samples of mice treated with AAV9-LacZ and -Wnt11 4 and 8 weeks after MI induction (Fig. 3A,B). This observation indicates that Wnt11 does not induce cardiac differentiation of bone marrow derived cells.

Furthermore, we examined other effects of Wnt11 on apoptosis, angiogenesis and hypertrophy of cardiomyocytes in infarcted heart. TUNEL staining indicates that Wnt11 does not affect apoptosis in border area 24 hours after MI induction (Suppl. Fig. 3A). CD31 staining to evaluate vascular density showed that Wnt11 has no effect on angiogenesis in border area and remote area (Suppl. Fig. 3B). We also evaluated cross sectional area of myocytes in border and remote area on day 28 and 56 after MI induction. Wnt11 did not induce hypertrophy of cardiomyocytes (Suppl. Fig. 3C). These data indicate that benefitial effects of Wnt11 can be exclusively attribute to suppressed incorporation of inflammatory cells after MI.

### Systemic rAAV9-Wnt11 administration suppresses the expression of inflammatory cytokines after MI

**Figure 5:**
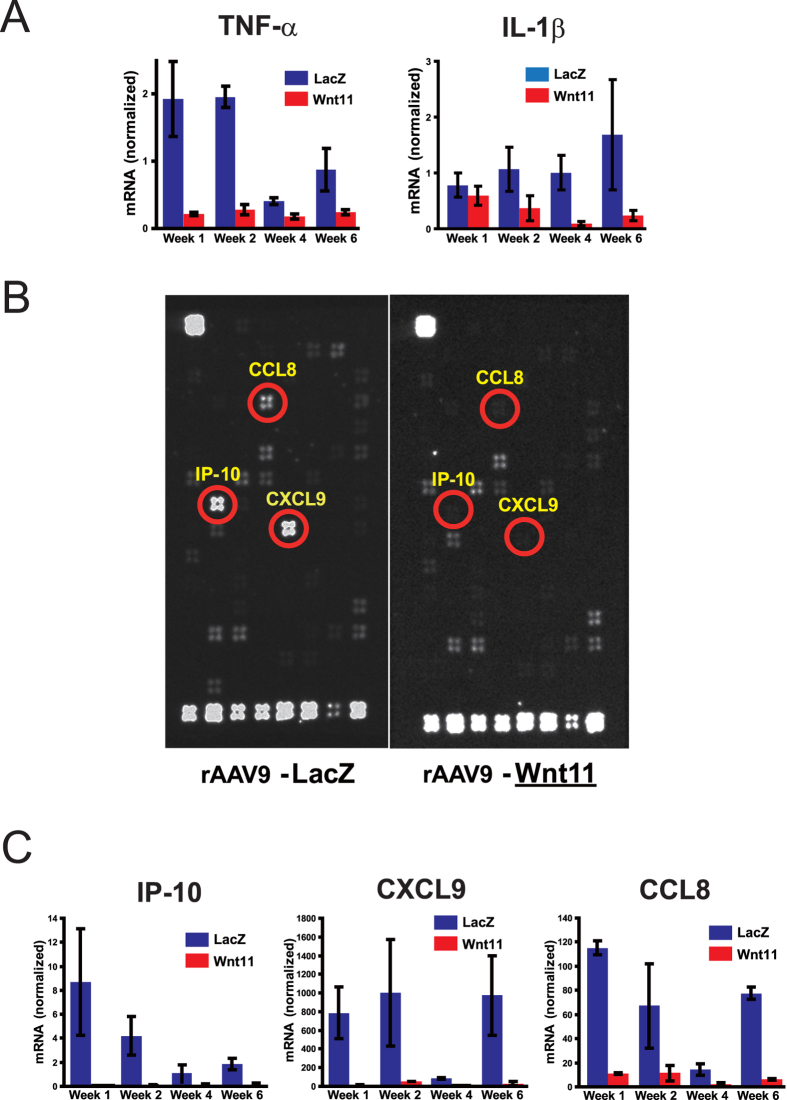
Wnt11 Expression during Recovery from MI Reduces the Expression of Inflammatory Factors. (**A**) From 1 to 6 weeks after MI, mRNA levels of TNF-α and IL-1β were measured via RT-PCR and normalized to 18s rRNA expression; n = 2 per group. (**B**) One week after MI gene array analyses were performed to identify inflammatory factors that may have been downregulated by Wnt11 expression. The array locations for IP-10, CXCL9, and CCL8 are circled in red. (**C**) From 1 to 6 weeks after MI, mRNA levels of the inflammatory factors IP-10, CXCL9, and CCL8 were measured via RT-PCR and normalized to 18S rRNA expression; n = 2 per treatment group.

To determine whether the impaired inflammatory-cell incorporation associated with rAAV9-Wnt11 administration was accompanied by decreased expression of inflammatory genes, mRNA expression of TNFα and IL-1β was measured by RT-PCR from 1 to 6 weeks after MI. At each time point, both genes were expressed at lower levels in rAAV9-Wnt11-treated mice than in mice treated with rAAV9-LacZ (Fig. 5A). Because TNFα induces the expression of many inflammatory cytokines^5^, gene array analyses were performed with samples collected 1 week after MI to identify other factors that may have been altered by the Wnt11 expression. Suppression of IP-10, CXCL9, and CCL8 expression was particularly dramatic (Fig. 5B) and was confirmed by RT-PCR analysis: from 1 to 6 weeks after MI, all three genes were expressed at much lower levels in rAAV9-Wnt11-treated mice than in rAAV9-LacZ-treated mice (Fig. 5C). Collectively, these findings suggest that Wnt11 gene expression improves survival and cardiac performance after MI by, at least in part, suppressing the inflammatory response.

We also examined gene expression of all Wnt proteins in mice treated with rAAV9-LacZ or rAAV9-Wnt11 after MI induction (Suppl. Fig. 4). Wnt11 did not significantly modulate gene expression of some Wnts which were induced in infarcted heart.

### Systemic rAAV9-Wnt11 administration shows similar effects to total body irradiation for myelosuppression

rAAV9-Wnt11 administration suppressed the incorporation of bone-marrow-derived cells in infarcted heart. We speculate that therapeutic effects of Wnt11 is attributed to the suppression of inflammatory cell infiltration. To confirm the influence of inflammatory cells in infarcted heart, we examined the effects of myelosuppression to reduce circulating white blood cells in myocardial infarction model. Total body irradiation (TBI) acutely induces hypoplasia in bone marrow. White blood cells (WBC) count in peripheral blood also reduced in a few days after TBI. Our preliminary data indicated that a dose of 3 Gy TBI induced leukopenia in mice. WBC count dramatically reduced within a day and reached to nadir by 4 days after TBI. The nadir continued for about one week and WBC count was gradually recovered. So we added second irradiation on day 10 after first irradiation. It resulted in long-lasting WBC suppression. Peripheral WBC count was still low on day 28 after first irradiation (Suppl. Fig. 5A). However, count of red blood cells showed minimum reduction in this protocol of irradiation. Platelet count was modestly reduced without bleeding tendency.

Next we examined the effects of total body irradiation (TBI) on myocardial infarction. We compared TBI mice, which had irradiation twice; 3 Gy immediately after MI induction and on day 10, with non-TBI mice. All mice in both groups were injected with 3.0 × 10^11^ GC of rAAV9-LacZ 1 week before MI induction. Surprisingly, all mice in TBI group survived for 8 weeks after MI induction (Suppl. Fig. 5B). Furthermore, sections in TBI group showed less infiltration of CD45 inflammatory cells than sections in non-TBI group 1 week after MI (Suppl. Fig. 5C).

Thus, effects by TBI in MI heart extremely resemble them by rAAV9-Wnt11. These data show that therapeutic effects of rAAV9-Wnt11 can be attributed to the suppression of inflammatory cells in infarcted heart more strongly.

### Wnt11 protein is produced by the stable transfectant cell line and released in the heparinized medium

**Figure 6:**
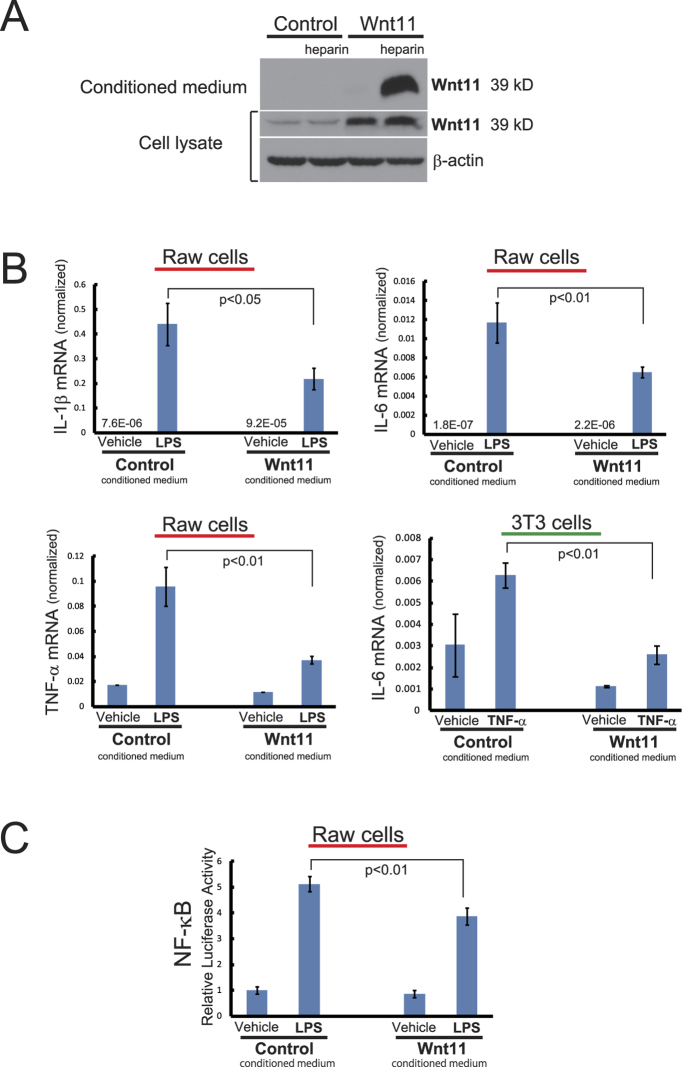
Conditioned Medium of Wnt11 Suppresses the Expression of Inflammatory Cytokines. (**A**) Conditioned media and cell lysates with or without 50 μg/mL heparin from HEK-control and HEK-Wnt11 clones were examined for expression of Wnt11 protein by Western blot. (**B**) Raw cells and 3T3 cells pretreated in the conditioned medium of HEK-control or HEK-Wnt11 were stimulated with 1 μg/mL of LPS and 20 ng/mL of TNF-α, respectively. RNA levels of IL-1β, IL-6 and TNF-α were measured via RT-PCR and normalized to β-actin. (**C**) After co-transfection of NF-κB luciferase and Renilla luciferase reporter plasmids, Raw cells were pretreated with the control or Wnt11 condition medium and then exposed to 1 μg/mL LPS for 8 hours. The relative luciferase activity was normalized to Renilla luciferase expression to adjust for variation in the transfection efficiency.

Clones of HEK 293 cells transfected with pEF6-Wnt11 vector were selected for the evaluation of Wnt11 protein expression. Conditioned media and cell lysates from clones cultured in plain DMEM with or without 50 μg/mL of heparin were examined for Wnt11 expression. HEK-Wnt11 clone transfected with pEF6-Wnt11 showed the expression of Wnt11 protein in the heparinized medium and cell lysate. Clone transfected with pEF6/Myc-His vector (HEK-control) was used as a control cell line. As shown in Fig. 6A, the band at approximately 39 kD was corresponding to mouse Wnt11 protein. Wnt proteins normally associate with cell membrane and extracellular matrix. Wnt11 protein produced by HEK-293 cells was dislodged from cell membrane and matrix into culture medium by the addition of heparin, which is a biochemical property of Wnt proteins (Fig. 6A). Conditioned media with 50 μg/mL of heparin from HEK-Wnt11 clone and HEK-control cells were used in the inflammation experiments *in vitro*.

### Wnt11 protein suppresses gene expression of inflammatory cytokines *in vitro*

Macrophage is a major cellular component of inflammatory reaction in myocardial infarction, generating pro-inflammatory cytokines such as IL-1, IL-6 and TNF-α that play central roles in the initiation and maintenance of inflammation. Effect of Wnt11 on the expression of inflammatory cytokines was examined using Raw 264.7 cells (mouse macrophage cell line) stimulated with LPS by quantitative RT-PCR. In response to LPS, gene expression of cytokines of IL-1, IL-6 and TNF-α was dramatically upregulated. However, Wnt11 conditioned medium significantly suppressed gene expression of these factors (Fig. 6B)

Next we performed quantitative PCR array analysis of inflammation-related genes to evaluate other inflammation-associated genes from Raw cells treated with control or Wnt11 conditioned medium before LPS stimulation. 36 factors of total 84 factors in the PCR array showed enough gene expression in either control or Wnt11 group to be compared. From the array analysis, no factor increased more than double in Wnt11 group. However, 10 factors decreased less than half in Wnt11 group compared to control group (Table 1). These data show that Wnt11 conditioned medium specifically suppresses gene expression of multiple inflammatory cytokines.

Fibroblast, cardiomyocyte and endothelial cell are important cellular components of heart tissue. We examined the effects of Wnt11 to NIH 3T3 cells (mouse fibroblast cell line), H9c2 cells (rat heart myoblast cell line) and HUVECs (human umbilical endothelial cells) stimulated with TNF-α. TNF-α increased gene expression of inflammatory cytokines from three cell lines. However, Wnt11 did not show significant effect on gene expression except for the suppression of IL-6 expression from NIH 3T3 cells (Fig. 6B).

### Wnt11 can modulate inflammatory reaction through the modulation of NF-κB

NF-κB is an important transcription factor that regulates cytokines and pro-inflammatory mediators such as TNF-α, IL-1 and IL-6 during inflammatory responses. We examined NF-κB-dependent promoter activity using a reporter gene assay to determine whether LPS-induced NF-κB activation might be regulated by Wnt11 protein. As shown in Fig. 6C, LPS increased NF-κB transcriptional level by 5-fold compared to the basal level. Moreover, LPS-induced activation of NF-κB was suppressed by the incubation in the conditioned medium of HEK-Wnt11 stable cell line. These data show that the suppression of inflammatory cytokines by Wnt11 conditioned medium can be induced via the regulation of NF-κB.

## Discussion

The inflammatory response is a double-edged sword for recovery from MI, and excessive or prolonged inflammation can worsen cardiac performance by promoting interstitial fibrosis in both infarcted and non-infarcted regions of the myocardium^4^,^5^. Although the link between the Wnt signaling and inflammation is controversial^12^, Wnt proteins appear to activate peripheral immune response^25^, to regulate interleukin production in monocytes and macrophages^15^,^16^, and to influence the development of lymphocytes^26^ and the fate of hematopoietic stem cells^13^.

Recently Wnt11 which is one of key players in heart development was reported to modulate inflammation by bacterial invasion in intestinal cells and myocarditis heart^17^,^18^,^20^. These reports show the possibility that modulation of Wnt proteins including Wnt11 could be potential new therapy for human diseases related to inflammation.

Here, we investigated whether the therapeutic overexpression of Wnt11 could alter the inflammatory response and improve survival and cardiac function after MI. Surprisingly, all rAAV9-Wnt11-treated mice survived until sacrifice at week 8 after MI, while more than 40% of mice treated with rAAV9-LacZ died during the same period. Wnt11 expression was also associated with significant improvements in cardiac function and appeared to reduce the incorporation of inflammatory cells and fibrosis in infarcted heart. Collectively, we demonstrate for the first time that Wnt11 expression regulates inflammation and improves recovery after myocardial injury.

The suppressed inflammatory response observed in rAAV9-Wnt11-treated mice was both broad-based and durable. One week after infarction, inflammatory (i.e., CD45+ and CD68+) cells were less common in hearts from rAAV9-Wnt11-treated mice than in hearts from mice treated with rAAV9-LacZ, while the incorporation of bone-marrow-derived (i.e., GFP+) cells and the expression of several inflammatory factors were reduced for up to 8 weeks. These effects likely contributed to the less extensive fibrosis associated with Wnt11 expression and reduced mortality by preventing the pleural effusion observed in deceased rAAV9-LacZ-treated mice. The difference of LVEF between Wnt11-treated mice and survived LacZ-treated mice is statistically significant, but small (Fig. 2B). However, even small difference of LVEF is critical for survival in MI mice which show LVEF less than 30% with huge infarction area on day1. Stroke volume (SV) recovered more efficiently in rAAV9- Wnt11-treated mice with time than in rAAV9-LacZ-treated mice. We consider that this significant improvement of SV in rAAV9-Wnt11-treated mice was attributed to compensation by non-infarction area where wall motion improved through the suppression of inflammation by Wnt11^3^. Furthermore, we compared the phenotype of Wnt11 treatment with effects by myelosuppression. Intriguingly, myelosuppression by total body irradiation significantly improved survival and decreased infiltration of CD45 positive cells in MI heart. The effects of myelosuppression in MI heart were very similar to the therapeutic effects of Wnt11 treatment which suppressed infiltration of multiple kinds of white blood cells. We expect that Wnt11 protein in heart tissue modulates inflammatory cells mobilized from bone marrow from these data.

On the other hand, Wnt11 regulates cardiac differentiation in embryonic development and induces cardiomyogenic differentiation of bone marrow mononuclear cells^27^. In our experiment, Wnt11 did not promote cardiac differentiation of mobilized bone marrow cells which were labeled with GFP, though we never deny the possibility that Wnt11 induces specific differentiation of resident premature- or stem-cells in infarcted heart. Moreover, Wnt11 did not modulate apoptosis, angiogenesis and hypertrophy of myocytes. These data indicate that suppression of inflammatory reaction by Wnt11 is considered to be main effect to improve survival of MI mice.

Macrophages play key roles in regulating phagocytosis of necrotic cells and inflammatory response to myocardial infarction^28^. Wnt11 conditioned medium reduced the gene expression of inflammatory cytokines through the modulation of NF-κB of Raw cells, macrophage cell line, stimulated with LPS. This effect indicates that Wnt11 protein could suppress inflammation in infarcted heart by regulating inflammatory cytokines from macrophages mobilized into the heart.

Wild-type AAVs have never been linked to tumor development or acute pathologies in humans^29^, which suggests that the vector-related safety of rAAV9-Wnt11 treatment should be acceptable for future clinical application. AAV1 and AAV6 have been investigated for treatment of heart failure in clinical trials^30^, but to our knowledge, the potential benefits of gene delivery with the rAAV9 system have not been previously characterized clinically. Our results confirm that rAAV9-mediated gene delivery is efficient, cardiac-specific, and durable: 2 weeks after administration, transgene expression was robust in the heart, much lower in the liver, and nearly undetectable elsewhere, and cardiac transgene levels remained high throughout the 8-week evaluation period.

In conclusion, systemic gene therapy with the rAAV9 vector induced robust, durable, and cardiac-specific transgene expression, and rAAV9-mediated Wnt11 expression was associated with significantly greater cardiac performance, significantly less fibrosis, and profound improvement in survival after MI, presumably via suppressed infiltration of inflammatory cells including macrophages and suppressed gene expression of inflammatory cytokines.

Thus, the rAAV9 system is a promising method for delivering genes to the heart, and therapies that modulate Wnt11 signaling could be effective therapeutic modality for MI and other diseases that are accompanied by inflammation. Finally, our data reveal a novel function of Wnt11 in the regulation of inflammatory response and provide a rationale for the use of Wnt11 to manipulate human diseases that are mediated by inflammation.

### Study limitation

In this study, rAAV9 vector was injected one week before MI induction to induce enough gene expression from early phase to subacute phase of myocardial infarction. It is impossible to apply vector before the onset of myocardial infarction clinically. However, we clearly showed the beneficial therapeutic effects of Wnt11 for myocardial infarction. We expect to be able to apply modified rAAV9 vector with a property of rapid gene expression or recombinant protein of Wnt11 with high bioactivity for Wn11 therapy in the near future.

## Methods

### Mice

Experiments with uninjured mice were conducted in C57BL/6J mice, and MI was surgically induced in male C57BL/6J mice or female C57BL/6J mice transplanted with bone marrow from male C57BL/6-Tg (CAG-EGFP)1Osb/J mice^31^. All experimental procedures and animal-care protocols were approved by the Institutional Animal Care and Use Committee of Northwestern University and Nagoya University. All experiments were carried out in accordance with the approved protocols and guidelines.

### Bone-marrow transplantation

Donor mice were 8 to 10 weeks of age and recipient mice were 5 weeks of age. The transplantation procedure was performed as described previously^32^. The transplanted bone marrow was allowed to regenerate for 6 weeks before MI was induced.

### Myocardial infarction

MI was induced as described previously^33^. The left-anterior–descending (LAD) branch of the left coronary artery was ligated proximal to the bifurcation between the LAD and the diagonal branch with 8-0 polypropylene sutures.

### AAV infection

The rAAV9 vectors which contain CMV promoter for high-level gene expression were provided by the Vector Core of the University of Pennsylvania via the 3 plasmids co-transfection method^34^ with modifications. For assessments performed in uninjured mice, 1 × 10^11^- or 3 × 10^11^-genome copy (GC) doses of rAAV9-LacZ or 3 × 10^11^-GC doses of rAAV9-Wnt11 were administered. For experiments performed in the MI model, 3 × 10^11^-GC doses of rAAV9-Wnt11 or rAAV9-LacZ were administered 1 week before MI was induced. Viruses were administered in 100 μL saline via tail-vein injection.

### Cardiac functional assessments

Cardiac function was evaluated with a high-resolution echocardiographic system (VEVO700, VisualSonics Inc.). Images were evaluated by planimetry, as recommended^35^. The endocardial area of each frame was calculated by tracing the endocardial limits of each frame in the long-axis view, then the minimal and maximal areas were designated as the left-ventricular end-systolic and end-diastolic volumes (LVESV and LVEDV), respectively. The system software uses a formula based on a cylindrical-hemiellipsoid model (volume = 8.area^2^/3π/length). Left-ventricular ejection fractions (LVEFs) and stroke volumes (SVs) were calculated from the following formulas:^36^ LVEF = (LVEDV – LVESV)/LVEDV × 100; SV = (aortic velocity time integral) × π (aortic valve diameter/2)^2^. LVEF and SV were calculated for three cardiac cycles per mouse per time point, and the average of the three cycles was recorded.

### Histological assessments

For assessments of β-galactosidase expression, organs were fixed with 0.2% glutaraldehyde at 4 °C for 4 hours, snap-frozen in O.C.T. compound. Sections were stained with X-gal as previously described^37^ and briefly counterstained with eosin. Fibrosis was evaluated by treating sections with 0.02% picrosirius red (Direct Red 80; Sigma-Aldrich Corp.) solution for 1 hour as previously described^38^. Perivascular fibrosis was assessed by calculating the ratio of the area of collagen-stained fibrosis to total vessel area. To identify incorporated bone-marrow-derived cells, the heart was perfused with 10% formalin and embedded in paraffin and sectioned. Sections were stained with rabbit polyclonal anti-GFP antibodies (Invitrogen Corporation) and mouse monoclonal anti-α-sarcomeric actin antibodies (clone 5C5; Sigma-Aldrich Corp.); then, the anti-GFP antibodies were labeled with Alexa-Fluor 488 secondary antibodies (Invitrogen Corporation), the anti-α-sarcomeric actin antibodies were labeled with Cy3 anti-mouse IgM secondary antibodies (Jackson ImmunoResearch Laboratories, Inc.), and sections were counterstained with DAPI. To evaluate CD45, CD68, CD3 and Ly6G expression, hearts were snap-frozen in O.C.T. compound. Sections were fixed with 4% paraformaldehyde, and then stained with anti-CD45 (BD Pharmingen), anti-CD68 (Serotec), anti-CD3 (Abcam) or anti-Ly6G (BD Pharmingen) primary antibodies and Alexa-Fluor-conjugated secondary antibodies (Invitrogen Corporation); nuclei were counterstained with DAPI. To evaluate vascular density, section were stained with anti-CD31 antibody (Invitrogen Corporation) after fixation by 4% paraformaldehyde. To assess apoptosis in the border area, TUNEL (Terminal deoxynucleotidyl-transferase-mediated dUTP Nick-End Labeling) assay was performed on the sections according to the instructions of the manufacturer (Roche). Myocyte size was examined by hematoxylin and eosin (H & E) staining.

### FACS (Fluorescence activated cell sorter) analyses of cardiac cells

Mice were sacrificed at day 7 after MI induction. The hearts were minced with fine scissors, and incubated in a cocktail of 0.1% collagenase B (Roche) and 2.2 U/mL Dispase II (Roche) at 37 °C for 30 minutes. Red blood cells were lysed using RBC lysis buffer (eBioscience). The samples were then filtered through a nylon mesh (40μm)^39^.

Rat serum (Sigma) and Purified Rat Anti-Mouse CD16/CD32 (BD Pharmingen) were added to the cells. Then the cells were incubated with FITC-conjugated Rat anti-Mouse CD45 antibody (BD Pharmingen) or APC-conjugated Rat anti-Mouse CD68 antibody (BioLegend). FITC Rat IgG2bk isotype (BD Pharmingen) or APC-Rat IgG2ak isotype (BioLegend) were used as a control for the anti-CD45 antibody and anti-CD68 antibody, respectively. For the staining with CD 68 antibody the cells were fixed with 0.01% formaldehyde and permeabilized by polyoxyethylene (20) sorbitan monolaurate (Wako) before blocking. Stained cells were diluted with PBS supplemented with 1% FBS before flow analysis of FACS Calibur (BD Biosciences).

### Real-time, reverse transcription polymerase chain reaction (RT-PCR) and gene array analyses

RNA was isolated from homogenized organ tissues of mice by using STAT-60 (TEL-TEST, Inc.), and total cellular RNA of cell lines was isolated with the RNeasy Plus Mini Kit (QIAGEN). For quantitative RT-PCR analyses, total RNA was reverse transcribed with a Taqman cDNA Synthesis Kit (Applied Biosystems) and a SuperScript First-Strand Synthesis System (Invitrogen), and amplification was performed on a Taqman 7500 (Applied Biosystems) and an Mx3000p QPCR system (Stratagene) for the examination of mouse samples and cell lines, respectively. Primer and probe sequences are reported in Supplemental Table 1, Table 2 and Table 3. The relative expression of each mRNA was calculated by the comparative threshold cycle method, and normalized to 18S rRNA expression for mouse samples and β-actin for cell line samples.

### Total body irradiation (TBI)

Mice were irradiated with 3 Gy TBI using an X-ray generator (MBR-1520A-3; Hitachi Medical Cooperation) twice immediately after MI induction and 10 days later. We followed these mice for 56 days after MI induction.

### Construction of plasmids and stable cell lines

The cDNA encoding mouse Wnt11 (Open Biosystems, Inc) ending with a stop codon was subcloned into pEF6/Myc-His B (Invitrogen) before sequence of Myc-His to obtain the pEF6-Wnt11. HEK 293 (ATCC) cells were grown in high glucose Dulbecco’s modified Eagle’s medium supplemented with 10% FBS and antibiotics (complete DMEM). Then the linearized plasmids of pEF6-Wnt11 and pEF6/Myc-His were separately transfected into the HEK 293 cells by Lipofectamine 2000 (Invitrogen), following the manufactory’s instruction. Transfected cells were diluted and cultured in the medium containing 10 μg/mL Blasticidin (Invitrogen). After 17 days later, the colonies resistant to Blasticidin were selected and transferred to 6 well-plates and cultured for expansion^40^.

### Preparation of conditioned media

Conditioned media were harvested from confluent cultures of either stable cell line of control or Wnt11, grown in complete DMEM with 50 μg/mL of heparin. Conditioned medium containing Wnt11 protein was obtained by solubilizing extracellular matrix- and cell-membrane-associated Wnt11 with the addition of 50 μg/mL heparin to confluent cultures;^41^ and the media were collected after 48-hour incubation.

### Western blot

Proteins from heart samples and colonies of transfected HEK 293 cells were subjected to electrophoresis on 10% polyacrylamide SDS gel and transferred onto polyvinylidene difluoride membrane, and the membrane was preincubated in 5% nonfat milk prior to incubation overnight at 4 °C with Wnt11 antibody (abcam) diluted 1:1000 in PBS containing 1% BSA. The membrane was incubated with horseradish peroxidase-conjugated secondary antibody. The signal was detected using ECL Western Blotting Detection Reagents (Amersham)^42^.

### Induction of inflammation by LPS or TNF –α in cell lines

Raw 264.7 (mouse macrophage cell line), NIH 3T3 (mouse embryonic fibroblast cell line), and H9c2 (rat heart myoblast cell line) (ATCC) were cultured in complete DMEM. Human umbilical vein endothelial cells (HUVECs) were cultured in EGM2 medium with 2% FBS in the standard fashion. The media of Raw, 3T3, H9c2 cells and HUVECs were replaced with conditioned media of Wnt11 or control stable cell lines followed by incubation of 2.5 hours. Raw cells were stimulated with 1 μg/mL of lipopolysaccharide (LPS; Escherichia coli, O111:B4) and incubated for 7 hours. 3T3, H9c2 cells and HUVECs were stimulated with 20 ng/mL of human TNF-α and incubated for 4 hours.

Gene array analyses of mouse samples were performed with 3 μg total RNA by using the Mouse Inflammatory Cytokines and Receptors GEArray (SABiosciences Corporation) as directed by the manufacturer’s instructions^43^. Expression of inflammatory genes in stimulated Raw cells was evaluated with Mouse Inflammatory Cytokines and Receptors RT^2^ Profiler PCR Array (SABiosciences; catalog #PAMM-011). The resulting threshold cycle values for all wells were analyzed using the RT^2^ Profiler PCR Array Data Analysis Template file provided by SABiosciences^44^.

### NF-κB reporter assay

Raw 264.7 cells were co-transfected with a pGL-κB-Luc vector (Promega) and a pGL-TK-Rluc control plasmid (Promega) using Lipofectamine 2000. The cells were pretreated with control or Wnt11 conditioned media for 90 minutes and then stimulated with 1 μg/mL of LPS for 8 hours. Following LPS treatment, luciferase activity of lysed cells was assessed using a dual-luciferase reporter assay system (Promega) and a luminomiter (TECAN Infinite M200) according the manufacturer’s protocols. The relative Firefly luciferase activity was calculated after normalization with Renilla reniformis activity to adjust for variation in the transfection efficiency^45^.

### Statistical analysis

Survival was summarized via Kaplan-Meier analysis and evaluated for significance with the log-rank test. Other results are presented as mean ± SEM; comparisons between two groups were evaluated for significance with the Student t-test, and measurements obtained in the same animal at multiple time points were evaluated via repeat measure analysis. A *P* value less than 0.05 was considered significant.

## Additional Information

**How to cite this article**: Morishita, Y. *et al.* Wnt11 Gene Therapy with Adeno-associated Virus 9 Improves Recovery from Myocardial Infarction by Modulating the Inflammatory Response. *Sci. Rep.* **6**, 21705; doi: 10.1038/srep21705 (2016).

## Supplementary Material

Supplementary Information

## Figures and Tables

**Table 1:** Wnt11 regulates expression of inflammatory cytokines and receptors in Raw 264.7 cells.

RefSeq	Symbol	Description	Fold Change
NM_013854	Abcf1	ATP-binding cassette, sub-family F (GCN20), member 1	0.9056
NM_009744	Bcl6	B-cell leukemia/lymphoma 6	0.4025
NM_009778	C3	Complement component 3	0.228
NM_009807	Casp1	Caspase 1	0.6404
NM_011333	Ccl2	Chemokine (C-C motif) ligand 2	0.4753
NM_009137	Ccl22	Chemokine (C-C motif) ligand 22	0.7749
NM_011337	Ccl3	Chemokine (C-C motif) ligand 3	0.5348
NM_013652	Ccl4	Chemokine (C-C motif) ligand 4	0.5893
NM_013653	Ccl5	Chemokine (C-C motif) ligand 5	0.4081
NM_013654	Ccl7	Chemokine (C-C motif) ligand 7	0.5441
NM_011338	Ccl9	Chemokine (C-C motif) ligand 9	0.8994
NM_009912	Ccr1	Chemokine (C-C motif) receptor 1	1.9145
NM_021274	Cxcl10	Chemokine (C-X-C motif) ligand 10	0.5366
NM_019494	Cxcl11	Chemokine (C-X-C motif) ligand 11	0.2916
NM_010548	Il10	Interleukin 10	0.5517
NM_008348	Il10ra	Interleukin 10 receptor, alpha	0.4374
NM_008349	Il10rb	Interleukin 10 receptor, beta	0.5536
NM_133990	Il13ra1	Interleukin 13 receptor, alpha 1	0.6699
NM_008357	Il15	Interleukin 15	0.2896
NM_008360	Il18	Interleukin 18	0.4299
NM_010554	Il1a	Interleukin 1 alpha	1.195
NM_008361	Il1b	Interleukin 1 beta	0.7255
NM_019450	Il1f6	Interleukin 1 family, member 6	1.5713
NM_013563	Il2rg	Interleukin 2 receptor, gamma chain	0.6294
NM_010559	Il6ra	Interleukin 6 receptor, alpha	1.0512
NM_008401	Itgam	Integrin alpha M	0.9474
NM_008404	Itgb2	Integrin beta 2	0.7695
NM_008518	Ltb	Lymphotoxin B	0.4284
NM_010798	Mif	Macrophage migration inhibitory factor	1.0331
NM_007926	Aimp1	Aminoacyl tRNA synthetase complex-interacting multifunctional protein 1	0.9573
NM_009263	Spp1	Secreted phosphoprotein 1	1.4211
NM_011577	Tgfb1	Transforming growth factor, beta 1	0.991
NM_013693	Tnf	Tumor necrosis factor	0.424
NM_011609	Tnfrsf1a	Tumor necrosis factor receptor superfamily, member 1a	0.8748
NM_011610	Tnfrsf1b	Tumor necrosis factor receptor superfamily, member 1b	0.7356
NM_023764	Tollip	Toll interacting protein	0.5771

Quantitative PCR array analysis of inflammatory cytokines and receptors in LPS-stimulated Raw 264.7 cells was performed to examine the effects of Wnt11. Data show gene expression in Wnt11 treated Raw cells compared to control Raw cells. Fold-down regulation is denoted in red.
