# Supplementary material for: Wnt11 Gene Therapy with Adeno-associated Virus 9 Improves Recovery from Myocardial Infarction by Modulating the Inflammatory Response
Source: Sci Rep. 2016 Feb 17;6:21705. doi: 10.1038/srep21705 (PMC4756373; doi:10.1038/srep21705)
Supplement: Supplementary Information [file srep21705-s1.pdf]

Wnt11 Gene Therapy with Adeno-associated Virus 9 Improves Recovery from Myocardial Infarction by Modulating the Inflammatory Response

Yoshihiro Morishita, Koichi Kobayashi, Ekaterina Klyachko, Kentaro Jujo, Kengo Maeda, Douglas W. Losordo, Toyoaki Murohara

Supplemental Material

Supplemental Figure Legends

Supplemental Figure 1. Endogenous Wnt11 is not Induced by Myocardial Infarction. 3.0×10^{11} GC of an rAAV9 vector coding for Wnt11 (rAAV9-Wnt11) or LacZ (rAAV9-LacZ) expression was injected into the tail vein of wild type (WT) mice transplanted with bone marrow from GFP-expressing mice. Endogenous expression of Wnt11 gene was measured after MI induction in WT mice. n=2 at each point.

Supplemental Figure 2. Wnt11 Expression Increases Cardiac Output and does not Change Left Ventricular End-diastolic Volume (LVEDV). Wnt11 Expression showed a tendency to decrease Left Ventricular End-systolic Volume (LVESV). One week before surgically induced MI, 3.0×10^{11} GC of a rAAV9 vector coding for Wnt11 (rAAV9-Wnt11) or LacZ (rAAV9-LacZ) expression was injected into the tail vein of mice. Echocardiographic assessment of cardiac output **(A)**, left ventricular end-diastolic volume (LVEDV) **(B)** and left ventricular end-systolic volume (LVESV) **(C)** was performed before injury and repeated in surviving mice on day 1 and weeks 2-8 after MI. rAAV-Wnt11: n=16 at each point, rAAV9-LacZ: n=16 Before MI and at Day 1 and Week 2; n=12 at Week 4; and n=9 at Week8. *P<0.001.

Supplemental Figure 3. Wnt11 Expression has no Effects on Apoptosis, Angiogeneis and Size of Cardiomyocytes. 3.0×10^{11} GC of a rAAV9 vector coding for Wnt11 (rAAV9-Wnt11) or LacZ (rAAV9-LacZ) expression was injected into the tail vein of wild type (WT) mice. **(A)** Apoptosis was evaluated by TUNEL staining (green) in the heart samples 24 hours after MI induction. n=3 per group. **(B)** Vascular density was evaluated

by staining sections of day 28 and day 56 for expression of CD31 (red) in border and remote area. n=3 per group **(C)** Cross sectional area of myocytes was evaluated in border and remote area by H&E staining. n=3 per group. (scale bar=100□m)

Supplemental Figure 4. Gene expression of all Wnt proteins in MI heart. Wnt11 did not significantly modulated gene expression of some Wnts which were induced in infarcted heart.

Supplemental Figure 5. Myelosuppression by Irradiation Decreases Infiltration of White Blood Cells in Infarcted Heart and Improves Survival, Which is the Similar Phenotype to Wnt11 Therapy. **(A)** The count of white blood cells in peripheral blood was followed for 8 weeks after two times of total body irradiation (TBI).n=2 at each point. **(B)** All mice in both groups (LacZ and LacZ-TBI) were injected with 3.0×10^{11} GC of rAAV9-LacZ 1 week before MI induction. Two times of 3 Gy irradiation just after MI and on day 10 were applied to mice of LacZ-TBI group. Survival was monitored for 8 weeks after MI; n=16 per group. **(C)** Infiltration of CD45 positive cells in the border zone and non-infarcted region were evaluated on day 7 after MI induction; scale bar=100mm.

Supplemental Figure 1

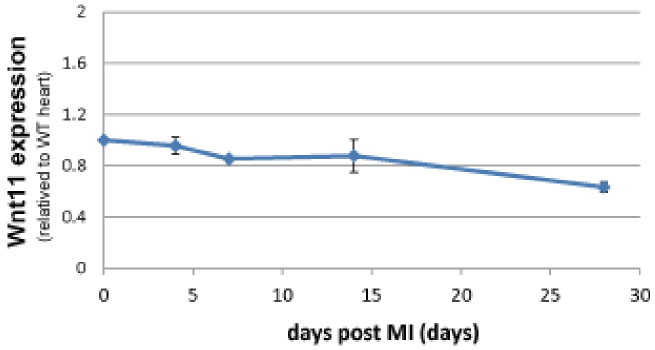

Supplemental Figure 2

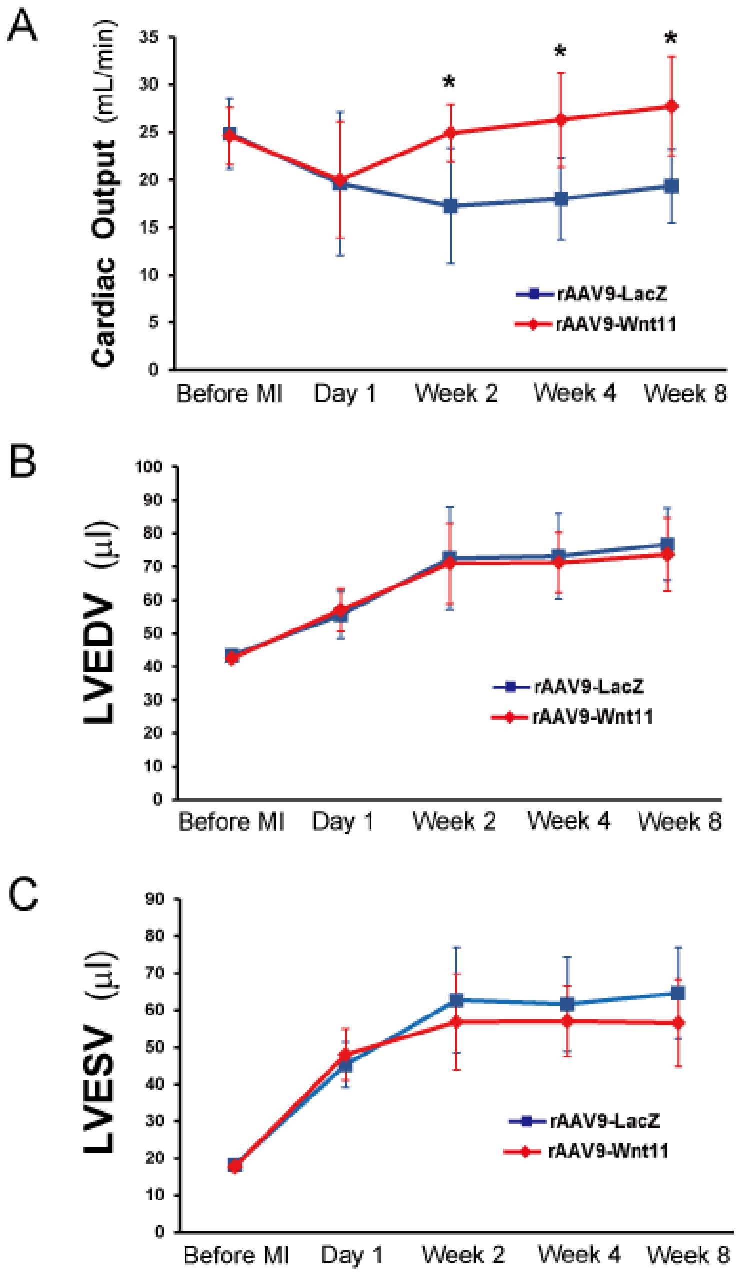

Supplemental Figure 3

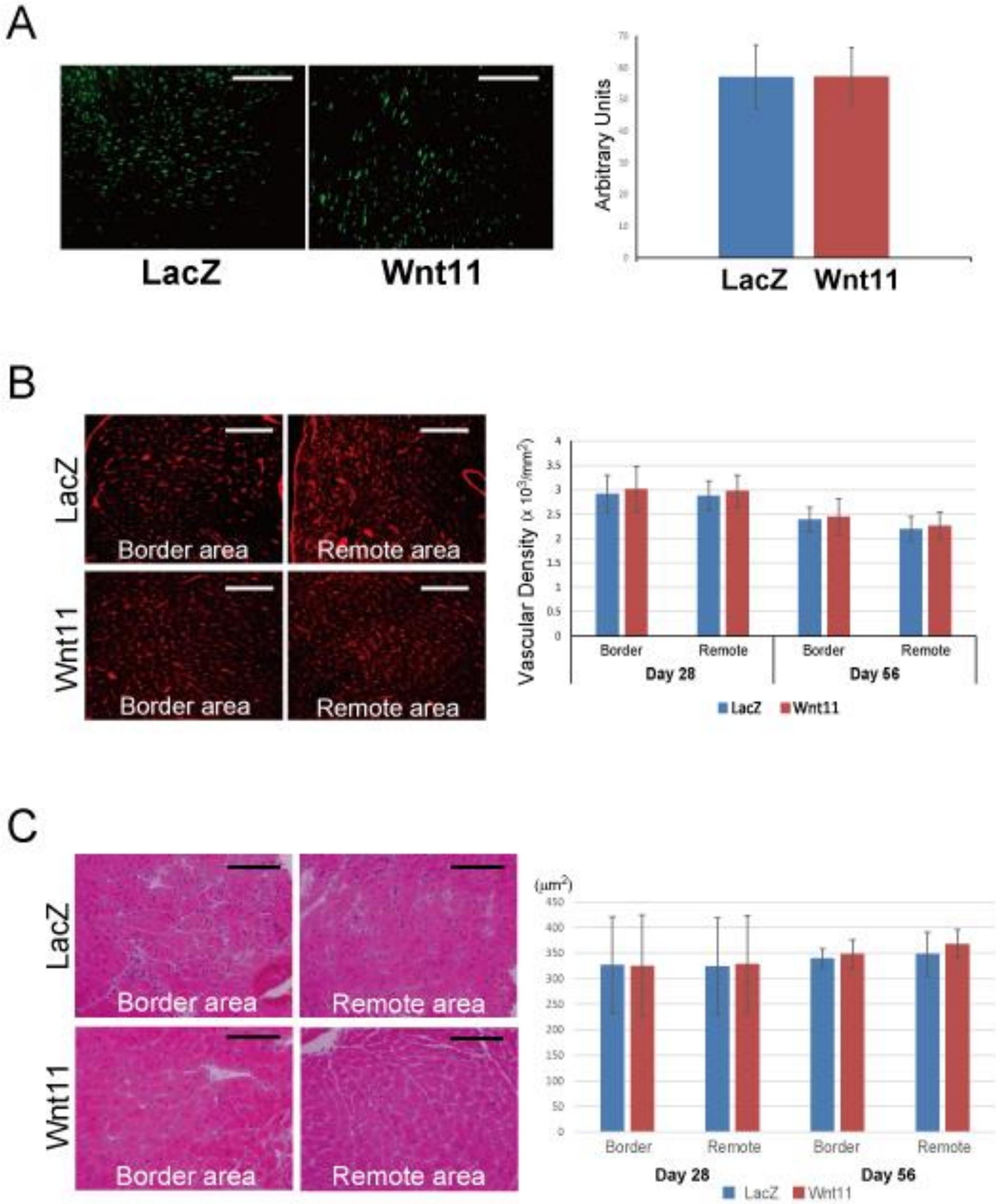

Supplemental Figure 4

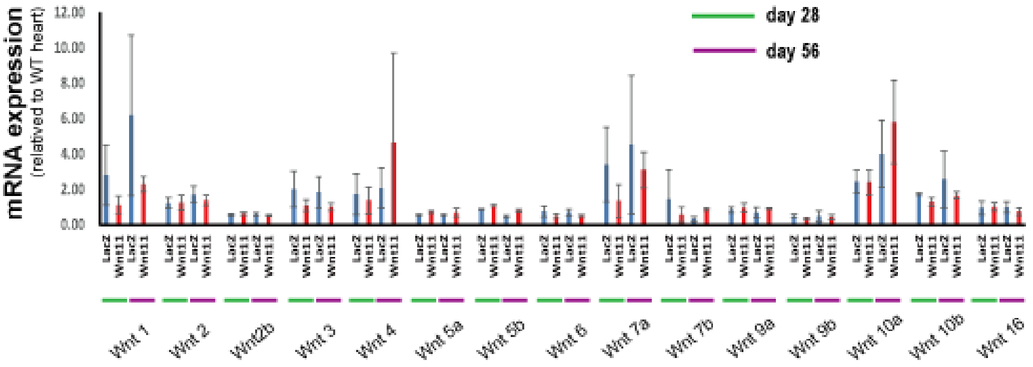

Supplemental Figure 5

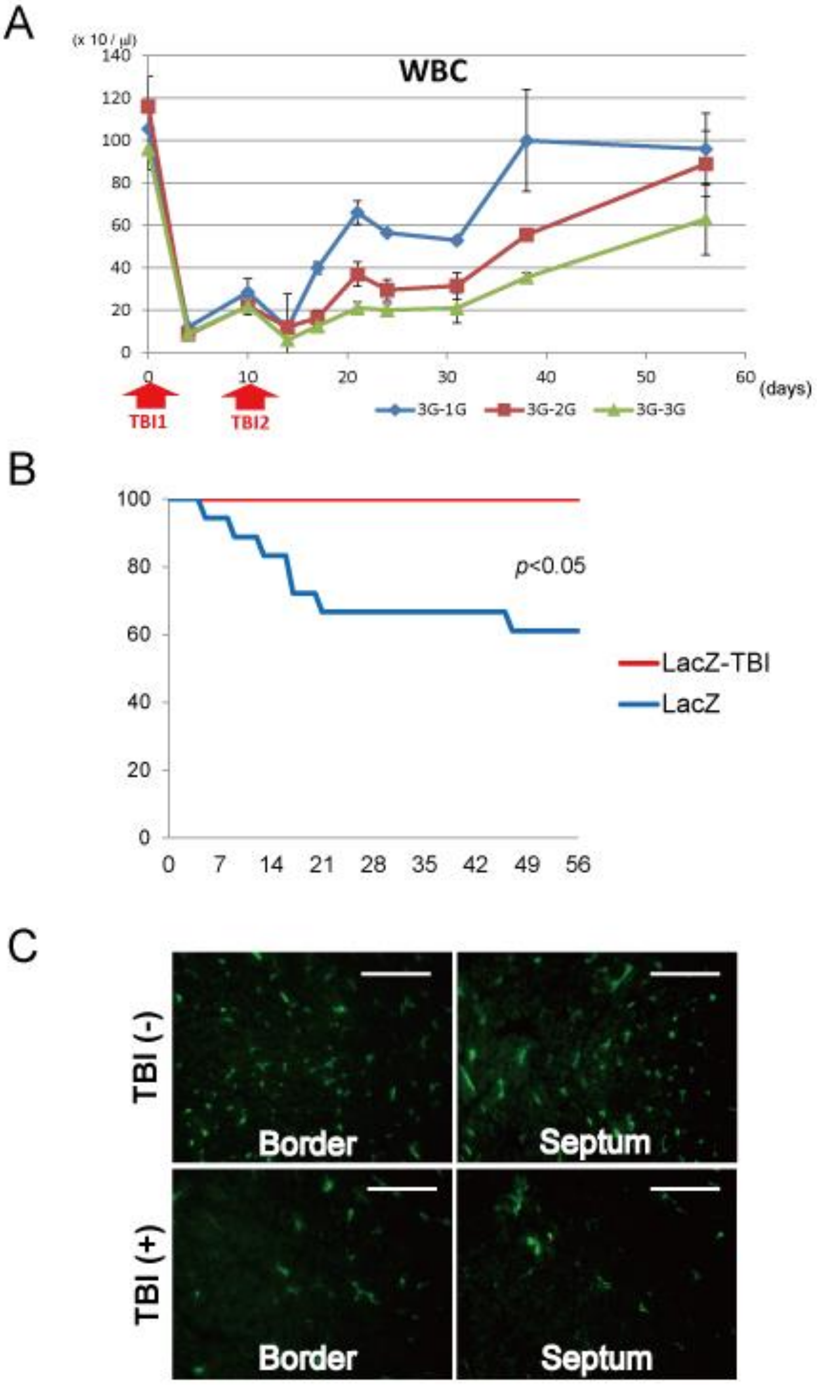

Supplemental Table 1. RT-PCR primers and probes for the examination of mouse samples (Taqman 7500)

Gene	Primers		Probe
	Forward	Reverse	
IP-10 (mouse)	GGACGGTCCGCTG CAA	CCCTATGGCCCTC ATTCTCA	TGCATCCATATCG ATGACGGGCC
lacZ	GGACGCGCGAATT GAATTA	CTGTTGACTGTAG CGGCTGATG	ACCAGTGGCGCGG CGACTTC
CCL8 (mouse)	TCCCTGTCAGCCC AGAGAAG	GGATATTGTTGAT TCTCTCGTAGCTTTT	AGCTGAAGATCCC CCTTCGGGTGC
CXCL9 (mouse)	AAATCCCTCAAAG ACCTCAAACAG	TCTTCAGTGTAGC AATGATTTCAGTTTT	TGCCCCAAGCCCC AATTGCA
IL-1β (mouse)	CTACAGGCTCCGA GATGAACAAC	TCCATTGAGGTGG AGAGCTTTC	AGCCTCGTGCTGT CGGACCCATATG
TNFα (mouse)	GGCTGCCCCGACT ACGT	AGGTTGACTTTCT CCTGGTATGAGA	CCTCACCCACACC GTCAGCCG
Wnt11 (mouse)	TCAGCCACGAAGG TGGTACA	CAGGTCCTTGGGC ACCAA	CGGCCTATGGGCA CCCGCA

Supplemental Table 2. RT-PCR primers for the examination of cell lines (Mx3000p)

	Primers	
**Gene**	**Forward**	**Reverse**
IL-1β (mouse)	TGAGCACCTTCTTTTCCTTCA	GGAGCCTGTAGTGCAGTTGTC
IL-1β (rat)	AGGAGAGACAAGCAACGACAA	TTGTTTGGGATCCACACTCTC
IL-1β (human)	GTGGCAATGAGGATGACTTGT	TGTAGTGGTGGTCGGAGATTC
IL-6 (mouse)	CAGAGGATACCACTCCCAACA	GAATTGCCATTGCACAACTCT
IL-6 (rat)	CAAGAGACTTCCAGCCAGTTG	TGGGTGGTATCCTCTGTGAAG
IL-6 (human)	CAGACAGCCACTCACCTCTTC	TTCAGGTTGTTTTCTGCCAGT
TNF-α (mouse)	GGTTCTCTTCAAGGGACAAGG	GGCAGAGAGGAGGTTGACTTT
TNF-α (rat)	CCTCAGCCTCTTCTCATTCCT	GGGAACTTCTCCTCCTTGTTG
TNF-α (human)	TAGCCCATGTTGTAGCAAACC	ATGAGGTACAGGCCCTCTGAT
GAPDH	AAGATTGTCAGCAATGCATCC	CTTCCACAATGCCAAAGTTGT

Supplemental Table 3. Wnt related RT-PCR primers for the examination of cell lines (Mx3000p)

Gene	Primers	
	Forward	Reverse
Wnt1 (mouse)	ATCCATCTCTCCCACCTCCTAC	GAATCTTTCTCTCACCCTCTGG
Wnt2 (mouse)	TCTGTCTATCTTGGGCATTCTG	TTCCTTCGCTATGTGATGTTTC
Wnt2b (mouse)	ACCTTCCTCTACCCTCAATCCT	TCACTCAGCCTCCTAAATCCAT
Wnt3 (mouse)	GTCTGCTAATGCTGGCTTGAC	TAGGAAGGGATGGGAGGTGT
Wnt3a (mouse)	TCGGAGATGGTGGTAGAGAAAC	TCGCAGAAGTTGGGTGAGG
Wnt4 (mouse)	AGAACTGGAGAAGTGTGGCTGT	AAAGGACTGTGAGAAGGCTACG
Wnt5a (mouse)	GTCCTTTGAGATGGGTGGTATC	ACCTCTGGGTTAGGGAGTGTCT
Wnt5b (mouse)	TGTCAGTTGTATCAGGAGCACA	GTGAAGGCAGTCTCTCGGCTA
Wnt6 (mouse)	TTTACACCAGCCCACGAAAG	ACTCACCCATCCATCCCAGTA
Wnt7a (mouse)	GGATGCTCACAGGGAAAGAAC	GCAGGAAACCCAGAATACCC
Wnt7b (mouse)	GCTCCTTCCTACTCGCTCTGT	GGTCCCTTTGTGGTTCACTTT
Wnt8a (mouse)	ACGGTGGAATTGTCCTGAGCATG	GATGGCAGCAGAGCGGATGG
Wnt8b (mouse)	GTTTGCTTGGGACCGTTG	TCCATTTCGGGAGTCATCA
Wnt9a (mouse)	ATGGTGTGTCTGGCTCCTG	CAGTGGCTTCATTGGTAGTGCT
Wnt9b (mouse)	GGGTGTGTGTGGTGACAATCT	GGTCCTTGCTTCCTCTCTTG
Wnt10a (mouse)	TCCTGTTCTTCCTACTGCTGCT	ACGCACACACACCTCCATC
Wnt10b (mouse)	CCACTACAGCCCAGAACCTC	GGAGAGACCCTTTCAACAACTG
Wnt16 (mouse)	GCTGTAACCTCCTCTGCTGTG	GTGGACATCGGTCATACTTTCA
